# Fracture risk and healthcare resource utilization and costs among osteoporosis patients with type 2 diabetes mellitus and without diabetes mellitus in Japan: retrospective analysis of a hospital claims database

**DOI:** 10.1186/s12891-016-1344-9

**Published:** 2016-11-25

**Authors:** Masayo Sato, Wenyu Ye, Tomoko Sugihara, Yoshitaka Isaka

**Affiliations:** 1 Medical Development Unit Japan, Eli Lilly Japan K.K, 7-1-5 Isogamidori, Chuo-ku, Kobe, Hyogo 651-0086 Japan; 2 Eli Lilly and Company, Indianapolis, IN USA; 3 inVentiv Health Clinical, Princeton, NJ USA

**Keywords:** Aged, Costs, Fracture, Healthcare resource utilization, Hospital claims database analysis, Japan, Osteoporosis, Raloxifene hydrochloride, Retrospective cohort study, Type 2 diabetes mellitus

## Abstract

**Background:**

Osteoporosis, osteoporosis-related fractures, and diabetes are considerable health burdens in Japan. Diabetes in patients with osteoporosis has been reported to be associated with increased fracture risk. This retrospective analysis of a Japanese hospital claims database investigated the real-world effect of type 2 diabetes mellitus (T2DM) on the incidence of clinical fractures, costs, and healthcare resource utilization in patients with osteoporosis and a subgroup of patients prescribed raloxifene.

**Methods:**

Women aged ≥50 years diagnosed with osteoporosis who had a first prescription claim for osteoporosis treatment with a pre-index period ≥12 months and a post-index period of 30 months were selected from a database extract (April 2008-July 2013). Patients prescribed raloxifene were classed as a subgroup. Patients diagnosed with T2DM constituted the T2DM group; all other patients (excluding patients with type 1 diabetes mellitus) constituted the non-diabetes mellitus (non-DM) group. Groups were matched by exact matching, using selected baseline characteristics. Patient demographic and clinical characteristics were compared using chi-squared tests, t-tests, or Wilcoxon rank sum tests. Time to first fracture was examined using Kaplan-Meier survival analysis.

**Results:**

Overall, the T2DM and non-DM groups had 7580 and 7979 patients, respectively; following matching, there were 3273 patients per group. In the raloxifene subgroup, the T2DM and non-DM groups had 668 and 699 patients, respectively; following matching, there were 239 patients per group. At baseline, the T2DM group (overall and raloxifene subgroup) had significantly higher healthcare resource utilization and comorbidities. During the post-index period, a similar pattern was observed in the overall group, even after matching; the T2DM group also had a higher incidence of fracture. In the raloxifene subgroup, after matching, there were no significant differences in fracture incidence or costs and fewer differences in healthcare resource utilization between the T2DM and non-DM groups.

**Conclusions:**

These findings suggest that comorbid T2DM increases fracture incidence in patients with osteoporosis, compared with patients without DM. Increases in fracture incidence were accompanied by greater costs and healthcare resource utilization, which are important considerations for clinical practice in Japan. Further research investigating the use of raloxifene for treatment of osteoporosis with comorbid T2DM may also be warranted.

**Electronic supplementary material:**

The online version of this article (doi:10.1186/s12891-016-1344-9) contains supplementary material, which is available to authorized users.

## Background

Osteoporosis and its resultant fractures are significant and expanding health burdens, impacting markedly on patient mortality, morbidity, and quality of life [1]. Given the prevalence of osteoporosis among the elderly, an enormous rise in its incidence has been predicted in Asia, where a 7.6-fold increase in the elderly population is expected [2]. By 2050, it is projected that 50% of all hip fractures will occur in Asia [2]. Japan currently has one of the world’s highest proportions of aged adults and this proportion is increasing; by 2050, 32% of the population is expected to be aged over 70 years [3]. The accompanying rise in the number of patients with osteoporosis is likely to have a major impact on healthcare resource utilization and costs. In Japan, treatment of hip fractures alone requires an average of 38.2 days in hospital and treatment costs per year are estimated to be 4.9 billion US dollars [3].

Effective management of osteoporotic fractures requires careful evaluation not only of osteoporosis but also of the increasing number of comorbidities that are thought to influence the risk of fractures [4]. Diabetes is emerging as one of the most important comorbidities in terms of its impact on fractures, as suggested by studies in the USA and in Europe [5, 6]. The presence of diabetes is independently associated with an increased risk of fracture, due in part to alterations in bone structure and strength [7]; the risk of hip fracture is almost twice as high for patients with diabetes than for those without diabetes [8]. The prevalence of diabetes, particularly type 2 diabetes mellitus (T2DM), in Japan is one of the highest in the world and has been increasing markedly, especially among the elderly [9, 10]. The rising prevalence of both osteoporosis and T2DM in the elderly Japanese population is likely to lead to greater numbers of patients with osteoporosis who also have TD2M.

Osteoporosis comorbid with T2DM may require a re-evaluation of diagnostic criteria or planned treatment; skeletal fragility due to T2DM can result even without a noticeable reduction in bone mineral density (BMD), which has traditionally been used to diagnose osteoporosis [7, 11]. Although not completely understood, this increased fragility has been proposed to be due to a deterioration of bone quality rather than bone mass [6, 7], and osteoporosis treatments that are able to address this deterioration may be preferred for patients with T2DM. In a subgroup analysis of the Multiple Outcomes of Raloxifene Evaluation study, for a small number of patients, Johnell and coauthors reported that the osteoporosis drug raloxifene showed higher efficacy in women with osteoporosis and diabetes than in women with osteoporosis [12]. A number of studies have suggested that raloxifene may prevent the deterioration of bone quality by reducing the formation of detrimental collagen cross-links [13] or by reducing inhibition of the Wnt/β-catenin signalling pathway, leading to bone formation and turnover [14, 15]. However, relatively little is known about whether raloxifene treatment has an impact on the incidence of fractures in osteoporosis patients with and without T2DM. There is a need for increased awareness of the potential impact of T2DM on osteoporotic fractures and how treatment costs, treatment patterns, and healthcare resource utilization in patients with osteoporosis may change depending on the presence or absence of T2DM. This information can help physicians optimize the treatment and management of patients with osteoporosis and T2DM.

The aim of this claims database study was to describe the real-world effect of T2DM comorbidity on the incidence of fracture, costs, and healthcare resource utilization in patients with osteoporosis in Japan. Furthermore, this study also aimed to describe the effect of raloxifene treatment on the incidence of fractures in patients with T2DM compared with patients without diabetes mellitus (DM).

## Methods

### Data source

This is a retrospective observational study using data extracted from a medical and pharmacy claims database provided by the Medical Data Vision Co. Ltd. (MDV; Tokyo, Japan). The MDV database comprises de-identified, longitudinal, patient-level medical and pharmacy claims from 121 hospitals in Japan; these hospitals represent 8% of all acute care hospitals in Japan. Data from more than 5.57 million unique patients, dating from April 2008 to July 2013, are contained in this extract of the MDV database.

### Study population

Women aged 50 years or older were included in the study if they had been diagnosed with osteoporosis, as indicated by International Classification of Diseases (10^th^ revision) (ICD-10) codes M80 (Osteoporosis with pathological fracture), M81 (Osteoporosis without pathological fracture), or M82 (Osteoporosis in diseases classified elsewhere) and they had a first prescription claim for osteoporosis medication from March 27, 2009 to February 12, 2011. The index date was defined as the date of the first prescription of any osteoporosis medication (within the MDV database extract corresponding to the period between April 2008 and July 2013). Other inclusion criteria were a pre-index period of at least 12 months (baseline period) and a post-index period of 30 months (follow-up period), which ensured that the original healthcare provider was accessed continuously for at least 42 months. Patients with type 1 diabetes mellitus (ICD-10 code E10), malnutrition-related diabetes mellitus (ICD-10 code E12), or secondary diabetes (ICD-10 code E13), or patients receiving antidiabetic medication without a diagnosis of T2DM during the study period were excluded from the study. Patients with osteoporosis were divided into two groups, depending on whether they had T2DM or did not have DM. Patients were classified as belonging to the T2DM group if they had been diagnosed with T2DM (during either the baseline period or the follow-up period), as indicated by ICD-10 codes E11 (Type 2 diabetes mellitus) or E14 (Unspecified diabetes mellitus). All other patients were classified as belonging to the non-DM group. Patients who initiated raloxifene treatment at the index date were identified as the raloxifene subgroup.

### Outcome measures

The outcome measures examined included the incidence of any clinical fracture as well as healthcare cost and resource utilization. Clinical fractures (identified from ICD-10 codes for fractures; Additional file 1: Table S1) included both clinical vertebral and non-vertebral fractures. Pathological fractures due to malignancies or pathologic bone processes (ICD-10 codes C00-D49) and traumatic fractures (see Additional file 1: Table S2) were excluded from the study. Recent fractures were defined as fractures occurring within the 6 months before the index date, whereas incident fractures were defined as new fractures occurring after the index date. The number of clinical fractures and non-vertebral fractures that occurred during the baseline period of the study were reported. In addition to baseline clinical fracture characteristics, the time from index date to first incident fracture was compared between the two groups during the 30-month follow-up period.

Healthcare resource utilization, which included osteoporosis-related tests and any laboratory tests, hospital admissions, and outpatient visits, as well as the number of days in hospital, and total costs (medical and pharmaceutical costs combined; Japanese Yen) during the baseline and follow-up periods were compared between the T2DM and non-DM groups. The proportion of patients who underwent tests for BMD, bone formation, and bone resorption were assessed separately and also as part of osteoporosis-related tests, which also included imaging tests.

### Statistical analysis

The demographics and clinical characteristics of patients with T2DM and without DM were compared using a chi-squared test for categorical variables and a *t*-test for all continuous variables except for cost. Cost was analyzed using a Wilcoxon Rank Sum Test. A *P* value <0.05 was considered statistically significant. The time to the first incident fracture was examined using a Kaplan-Meier survival curve analysis. To reduce the imbalance in covariates between groups, T2DM patients were matched 1:1 to non-DM patients using an exact matching method. The comparative analyses of patients with T2DM and without DM were repeated on the 1:1 matched samples. Groups were matched using selected baseline characteristics, which included age, name of osteoporosis drug at index date, gender, comorbidities (dyslipidemia, arteriosclerosis, peripheral vascular disease, thyroid disease, liver disease, and chronic kidney disease), other medications (corticosteroid, proton-pump inhibitor, thyroid hormone, anticonvulsants, and immunosuppressants), BMD tests, clinical fractures, recent fractures, non-vertebral fractures, and hospital admissions. These baseline characteristics were exactly matched, except in the case of age, for which a difference of 3 years (at most) was allowed between groups. Analyses were performed using SAS Version 9.2 (Cary, NC, USA).

## Results

### Patient disposition

A total of 233,166 patients in the database were identified as having a diagnosis of osteoporosis and having been prescribed medication for osteoporosis from March 27, 2009 to February 12, 2011. Of these patients, 15,559 met the eligibility criteria, with 7580 identified as also having T2DM; the remaining 7979 patients constituted the non-DM group. Of the patients who met the eligibility criteria, a total of 1367 patients (668 with T2DM, 699 without DM) were identified as having been prescribed raloxifene at the index date, including patients prescribed a combination of raloxifene and alendronate (28/1367 patients; Table 1).

**Table 1:** Age, fracture and clinical characteristics, and healthcare resource utilization at baseline

	Overall				Raloxifene subgroup			
Characteristic	All (*N* = 15,559)	T2DM (*N* = 7580)	Non-DM (*N* = 7979)	*P* value	All (*N* = 1367)	T2DM (*N* = 668)	Non-DM (*N* = 699)	*P* value
Age in years, mean (SD)	73.1 (9.10)	73.4 (8.99)	72.9 (9.19)	<0.001	73.7 (9.08)	74.9 (8.45)	72.6 (9.53)	<0.001
Age, n (%)				<0.001				<0.001
50–54 years	439 (2.8)	203 (2.7)	236 (3.0)		35 (2.6)	8 (1.2)	27 (3.9)	
55–59 years	896 (5.8)	413 (5.4)	483 (6.1)		62 (4.5)	22 (3.3)	40 (5.7)	
60–64 years	1515 (9.7)	679 (9.0)	836 (10.5)		114 (8.3)	40 (6.0)	74 (10.6)	
65–69 years	2309 (14.8)	1064 (14.0)	1245 (15.6)		229 (16.8)	103 (15.4)	126 (18.0)	
70–74 years	2967 (19.1)	1504 (19.8)	1463 (18.3)		264 (19.3)	144 (21.6)	120 (17.2)	
75–79 years	3406 (21.9)	1695 (22.4)	1711 (21.4)		280 (20.5)	145 (21.7)	135 (19.3)	
80 years and older	4027 (25.9)	2022 (26.7)	2005 (25.1)		383 (28.0)	206 (30.8)	177 (25.3)	
Fractures, n (%)								
Patients with clinical fractures^a^	810 (5.2)	405 (5.3)	405 (5.1)	0.453	76 (5.6)	45 (6.7)	31 (4.4)	0.063
Patients with non-vertebral fractures	542 (3.5)	275 (3.6)	267 (3.3)	0.338	62 (4.5)	33 (4.9)	29 (4.1)	0.482
Charlson Comorbidity Index, mean (SD)	2.15 (2.28)	2.76 (2.37)	1.58 (2.03)	<0.001	1.70 (1.87)	2.30 (1.99)	1.13 (1.55)	<0.001
Comorbidities, n (%)								
Dyslipidemia	5329 (34.3)	3671 (48.4)	1658 (20.8)	<0.001	429 (31.4)	323 (48.4)	106 (15.2)	<0.001
Arteriosclerosis	1487 (9.6)	935 (12.3)	552 (6.9)	<0.001	112 (8.2)	75 (11.2)	37 (5.3)	<0.001
Peripheral vascular disease	1926 (12.4)	1178 (15.5)	748 (9.4)	<0.001	164 (12.0)	96 (14.4)	68 (9.7)	0.008
Thyroid disease	1702 (10.9)	1120 (14.8)	582 (7.3)	<0.001	138 (10.1)	93 (13.9)	45 (6.4)	<0.001
Liver disease	2817 (18.1)	1914 (25.3)	903 (11.3)	<0.001	212 (15.5)	155 (23.2)	57 (8.2)	<0.001
Chronic kidney disease	823 (5.3)	647 (8.5)	176 (2.2)	<0.001	57 (4.2)	49 (7.3)	8 (1.1)	<0.001
Type 2 diabetes	4580 (29.4)	4580 (60.4)	-	<0.001	357 (26.1)	357 (53.4)	-	<0.001
Osteoporosis medications initiated, n (%)								
Bisphosphonates	8913 (57.3)	4140 (54.6)	4773 (59.8)	<0.001	47 (3.4)	18 (2.7)	29 (4.1)	0.140
SERMs	1373 (8.8)	670 (8.8)	703 (8.8)	0.950	1367 (100.0)	668 (100.0)	699 (100.0)	-
Active vitamin D^b^	6155 (39.6)	3101 (40.9)	3054 (38.3)	<0.001	385 (28.2)	167 (25.0)	218 (31.2)	0.011
Calcitonin	4 (0.0)	1 (0.0)	3 (0.0)	0.343^c^	-	-	-	
Raloxifene without alendronate	1339 (8.6)	660 (8.7)	679 (8.5)	0.661	1339 (98.0)	660 (98.8)	679 (97.1)	0.030
Raloxifene and alendronate in combination	28 (0.2)	8 (0.1)	20 (0.3)	0.033	28 (2.0)	8 (1.2)	20 (2.9)	0.030
Alendronate without raloxifene	5363 (34.5)	2476 (32.7)	2887 (36.2)	<0.001	-	-	-	-
Other medications, n (%)								
Corticosteroid	3020 (19.4)	1910 (25.2)	1110 (13.9)	<0.001	157 (11.5)	94 (14.1)	63 (9.0)	0.003
Proton-pump inhibitor	3035 (19.5)	2018 (26.6)	1017 (12.7)	<0.001	245 (17.9)	168 (25.1)	77 (11.0)	<0.001
Thyroid hormone	712 (4.6)	429 (5.7)	283 (3.5)	<0.001	41 (3.0)	25 (3.7)	16 (2.3)	0.115
Anticonvulsants	289 (1.9)	181 (2.4)	108 (1.4)	<0.001	21 (1.5)	15 (2.2)	6 (0.9)	0.037
Immunosuppressants	953 (6.1)	587 (7.7)	366 (4.6)	<0.001	51 (3.7)	29 (4.3)	22 (3.1)	0.244
Diabetes medications, n (%)								
Insulin	365 (2.3)	365 (4.8)	-	<0.001	29 (2.1)	29 (4.3)	-	<0.001
Metformin	249 (1.6)	249 (3.3)	-	<0.001	32 (2.3)	32 (4.8)	-	<0.001
Sulfonylurea	467 (3.0)	467 (6.2)	-	<0.001	50 (3.7)	50 (7.5)	-	<0.001
Alpha-glucosidase inhibitor	472 (3.0)	472 (6.2)	-	<0.001	48 (3.5)	48 (7.2)	-	<0.001
Thiazolidinedione	219 (1.4)	219 (2.9)	-	<0.001	25 (1.8)	25 (3.7)	-	<0.001
Resource utilization								
BMD test, n (%)	2251 (14.5)	913 (12.0)	1338 (16.8)	<0.001	203 (14.9)	71 (10.6)	132 (18.9)	<0.001
Bone formation test, n (%)	112 (0.7)	61 (0.8)	51 (0.6)	0.222	8 (0.6)	5 (0.7)	3 (0.4)	0.439^c^
Bone resorption test, n (%)	484 (3.1)	223 (2.9)	261 (3.3)	0.237	58 (4.2)	25 (3.7)	33 (4.7)	0.370
Osteoporosis-related tests^d^ (baseline), n (%)	3738 (24.0)	1752 (23.1)	1986 (24.9)	0.010	323 (23.6)	133 (19.9)	190 (27.2)	0.002
Osteoporosis-related tests^d^ (baseline), mean per patient (SD)	0.421 (0.980)	0.434 (1.041)	0.408 (0.918)	0.098	0.358 (0.823)	0.34 (0.859)	0.375 (0.788)	0.433
Laboratory tests, n (%)	1975 (12.7)	1199 (15.8)	776 (9.7)	<0.001	179 (13.1)	120 (18.0)	59 (8.4)	<0.001
Laboratory tests, mean per patient (SD)	8.983 (41.74)	13.02 (51.36)	5.144 (29.35)	<0.001	7.734 (34.43)	11.66 (41.1)	3.986 (26)	<0.001
Hospital admissions, n (%)	1410 (9.1)	872 (11.5)	538 (6.7)	<0.001	81 (5.9)	54 (8.1)	27 (3.9)	<0.001
Hospital admissions, mean per patient (SD)	0.117 (0.47)	0.147 (0.488)	0.088 (0.45)	<0.001	0.071 (0.313)	0.099 (0.378)	0.044 (0.232)	0.001
Days in hospital, mean (SD)	3.282 (21.65)	4.33 (27.37)	2.286 (14.15)	<0.001	1.688 (9.911)	2.147 (11.16)	1.25 (8.537)	0.097
Outpatient visits, n (%)	15,297 (98.3)	7437 (98.1)	7860 (98.5)	0.056	1353 (99.0)	660 (98.8)	693 (99.1)	0.533
Outpatient visits, mean per patient (SD)	3.567 (8.038)	4.216 (8.932)	2.95 (7.03)	<0.001	2.52 (3.836)	2.91 (4.524)	2.147 (2.992)	<0.001
Total cost in JPY, mean (SD)	122,000 (456,000)	160,000 (535,000)	85,215 (361,000)	<0.001	60,494 (261,000)	81,248 (325,000)	40,661 (178,000)	0.005
Total cost in JPY, median	13,983	18,675	10,783		9,565	13,066	6,654	

*Abbreviations*: *BMD* bone mineral density, *DM* diabetes mellitus, *JPY* Japanese Yen, *SD* standard deviation, *SERM* selective estrogen receptor modulator, *T2DM* type 2 diabetes mellitus

^a^Clinical fractures include vertebral and non-vertebral fractures

^b^Active vitamin D refers to calcitriol, alfacalcidol or eldecalcitol

^c^Chi-squared test may not be valid as more than 20% of the cells have fewer than 5 expected counts

^d^Osteoporosis-related tests include bone formation tests, bone resorption tests, imaging tests, and BMD measurements

### Age, fracture and clinical characteristics, and healthcare resource utilization at baseline (overall group)

The average ages of patients in the T2DM and non-DM groups were 73.4 and 72.9 years, respectively, with those aged 75 years or older constituting the largest proportion (>46%) of patients in both groups (Table 1); the average age of patients in the T2DM group was significantly higher than those in the non-DM group (*P* < 0.001). There were no significant differences in the fracture characteristics of patients in the T2DM and non-DM groups at baseline (Table 1). In terms of clinical characteristics, compared with the non-DM group, the T2DM group had a number of significant differences (Table 1), including a higher Charlson Comorbidity Index, a higher percentage of patients with comorbidities other than T2DM (including dyslipidemia, arteriosclerosis, peripheral vascular disease, thyroid disease, liver disease, and chronic kidney disease), a higher percentage of patients with active vitamin D (initiation of prescription), corticosteroid, proton-pump inhibitor, and diabetic medication prescriptions, and a lower percentage of patients with bisphosphonate (initiation of prescription) prescriptions (*P* < 0.001 for all characteristics).

A greater proportion of patients in the T2DM group utilized healthcare resources at baseline, when compared with the non-DM group (Table 1). The T2DM group had significantly higher total healthcare costs (*P* < 0.001), a higher percentage of patients with hospital admissions (*P* < 0.001), a higher average number of days spent in hospital (*P* < 0.001), and a higher percentage of patients undergoing laboratory tests (*P* < 0.001). In contrast, a significantly smaller percentage of patients in the T2DM group underwent osteoporosis-related tests (*P* = 0.010) or, more specifically, BMD tests (*P* < 0.001), compared with the non-DM group.

Following 1:1 exact matching, there were 3273 patients in both the T2DM and non-DM groups (data not shown). Comparison of the matched groups showed fewer significant differences between groups at baseline, with no differences in fracture characteristics. There were only a few significant differences in healthcare resource utilization, including higher total mean (54,931 JPY vs 42,338 JPY; *P* = 0.011) and median (12,441 JPY vs 9,841 JPY; *P* < 0.001) costs, as well as a higher percentage of patients undergoing laboratory tests (15.0% vs 9.8%; *P* < 0.001) in the T2DM group.

### Age, fracture and clinical characteristics, and healthcare resource utilization at baseline (raloxifene subgroup)

Within the raloxifene subgroup, the differences in fracture and clinical characteristics, and in healthcare resource utilization between the T2DM and non-DM groups showed a very similar pattern to that of the overall study group (Table 1). However, there were significant differences in some clinical characteristics that were observed in the overall study group but not in the raloxifene subgroup; there were no significant differences in the proportion of patients being prescribed bisphosphonates (initiation of prescription) and immunosuppressants, between the T2DM-raloxifene and non-DM-raloxifene subgroups (Table 1). In the raloxifene subgroup, there was a significantly higher percentage of non-DM patients who initiated active vitamin D (25.0% vs 31.2%; *P* = 0.011) prescriptions, in contrast to the overall study group, in which a higher percentage of T2DM patients initiated active vitamin D (40.9% vs 38.3%; *P* < 0.001) prescriptions.

Following 1:1 exact matching, there were 239 patients in both the T2DM and non-DM groups (data not shown). Similar to what was observed for the overall study group, the matched T2DM and non-DM groups were similar. The only remaining significant difference between the groups was the higher percentage of patients who underwent laboratory tests in the T2DM group (15.9% vs 6.7%; *P* = 0.001).

### Fracture characteristics and health resource utilization during the follow-up period (overall group)

Before 1:1 exact matching, the T2DM and non-DM groups showed statistically significant differences in almost all fracture and health resource utilization characteristics examined during the 30-month follow-up period (Table 2). The T2DM group had a significantly higher percentage of patients with clinical (*P* < 0.001) and non-vertebral (*P* < 0.001) fractures compared with the non-DM group. An analysis of the number of days to the first fracture in both groups showed that patients in the T2DM group were more likely to develop new fractures than those in the non-DM group (Table 2). These differences in fracture characteristics were not observed during the baseline (pre-matching) period (Table 1). However, the significant differences in healthcare resource utilization observed between the T2DM and non-DM groups during the follow-up period followed a similar pattern to what was observed during the baseline period (Tables 1 and 2).

**Table 2:** Comparison of fracture characteristics and healthcare resource utilization between groups (follow-up period; overall study group)

	Unadjusted				Matched			
Characteristic	All (*N* = 15,559)	T2DM (*N* = 7580)	Non-DM (*N* = 7979)	*P* value	All (*N* = 6546)	T2DM (*N* = 3273)	Non-DM (*N* = 3273)	*P* value
Fractures								
Patients with clinical fractures^a^, n (%)	1882 (12.1)	1029 (13.6)	853 (10.7)	<0.001	738 (11.3)	415 (12.7)	323 (9.9)	<0.001
Number of days to first fracture, mean (SD)	442.8 (259.0)	443.4 (257.7)	442.2 (260.6)	<0.0001^b^	454.5 (261.8)	455.5 (259.2)	453.3 (265.5)	0.0003^b^
Patients with non-vertebral fractures, n (%)	1454 (9.3)	817 (10.8)	637 (8.0)	<0.001	565 (8.6)	329 (10.1)	236 (7.2)	<0.001
Resource utilization								
BMD test, n (%)	6633 (42.6)	2739 (36.1)	3894 (48.8)	<0.001	2726 (41.6)	1271 (38.8)	1455 (44.5)	<0.001
Bone formation test, n (%)	768 (4.9)	384 (5.1)	384 (4.8)	0.466	279 (4.3)	135 (4.1)	144 (4.4)	0.582
Bone resorption test, n (%)	1535 (9.9)	634 (8.4)	901 (11.3)	<0.001	609 (9.3)	278 (8.5)	331 (10.1)	0.024
Osteoporosis-related tests^c^ (post-baseline), n (%)	11,454 (73.6)	5594 (73.8)	5860 (73.4)	0.614	4748 (72.5)	2430 (74.2)	2318 (70.8)	0.002
Osteoporosis-related tests^c^ (post-baseline), mean per patient (SD)	2.873 (3.198)	2.994 (3.369)	2.758 (3.022)	<0.001	2.719 (3.057)	2.86 (3.118)	2.578 (2.989)	<0.001
Hospital admissions, n (%)	4799 (30.8)	2980 (39.3)	1819 (22.8)	<0.001	1941 (29.7)	1177 (36.0)	764 (23.3)	<0.001
Hospital admissions, mean per patient (SD)	0.585 (1.451)	0.776 (1.646)	0.405 (1.212)	<0.001	0.515 (1.46)	0.635 (1.687)	0.395 (1.179)	<0.001
Days in hospital, mean (SD)	11.58 (36.23)	16.02 (44.22)	7.366 (25.8)	<0.001	9.622 (30.03)	12.33 (34.71)	6.914 (24.16)	<0.001
Outpatient visits, n (%)	15,511 (99.7)	7561 (99.7)	7950 (99.6)	0.205	6529 (99.7)	3270 (99.9)	3259 (99.6)	0.008
Outpatient visits, mean per patient (SD)	39.52 (54.6)	46.84 (58.98)	32.56 (49.09)	<0.001	36.26 (45.97)	40.06 (45.13)	32.45 (46.5)	<0.001
Laboratory tests, n (%)	3010 (19.3)	1645 (21.7)	1365 (17.1)	<0.001	1338 (20.4)	762 (23.3)	576 (17.6)	<0.001
Laboratory tests, mean per patient (SD)	52.11 (182.8)	68.84 (215.1)	36.21 (144)	<0.001	47.56 (157.9)	57.57 (167.1)	37.54 (147.5)	<0.001
Total cost in JPY, mean (SD)	1,130,000 (2,390,000)	1,490,000 (2,750,000)	782,000 (1,940,000)	<0.001	912,000 (1,860,000)	1,080,000 (1,930,000)	743,000 (1,770,000)	<0.001
Total cost in JPY, median	322,000	517,000	209,000		295,000	401,000	219,000	<0.001^d^

*Abbreviations*: *BMD* bone mineral density, *DM* diabetes mellitus, *JPY* Japanese Yen, *SD* standard deviation, *T2DM* type 2 diabetes mellitus

^a^Clinical fractures include vertebral and non-vertebral fractures

^b^ *P* value from Log Rank test

^c^Osteoporosis-related tests include bone formation tests, bone resorption tests, imaging tests, and BMD measurements

^d^ *P* value from Wilcoxon Rank Sum test

**Fig. 1:**
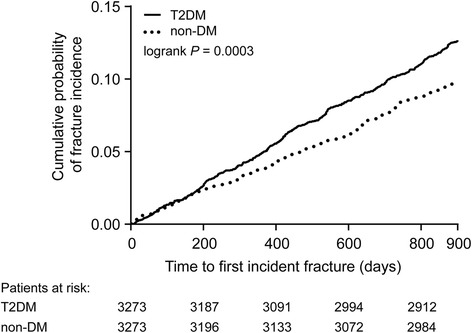
Kaplan-Meier curve of time to first incident fracture for the overall study group, post-matching. Abbreviations: DM = diabetes mellitus; T2DM = type 2 diabetes mellitus

Following 1:1 exact matching, an almost identical pattern of differences was observed in fracture characteristics and healthcare resource utilization of the T2DM and non-DM groups compared with characteristics before matching (Table 2). The only changes were in the number of patients who underwent osteoporosis-related tests during the follow-up period and the number of patients with outpatient visits, which were now significantly higher in the matched T2DM group than the matched non-DM group (*P* = 0.002 and 0.008, respectively). After group matching, the time to first incident fracture analysis showed that patients in the T2DM group were more likely to develop new fractures than those in the non-DM group (Fig. 1).

### Fracture characteristics and healthcare resource utilization during the follow-up period (raloxifene subgroup)

The significant differences in fracture characteristics and healthcare resource utilization observed between the T2DM and non-DM groups in the raloxifene subgroup during the follow-up period (Table 3) followed a similar pattern to that observed during the baseline (pre-matching) period (Table 1). However, in contrast to the baseline period, the T2DM group had a significantly higher number of days spent in hospital (*P* < 0.001), compared with the non-DM group (Table 3). Unlike the overall study group (Table 2), there were no statistically significant differences in the fracture characteristics of the T2DM-raloxifene group compared with the non-DM-raloxifene group (Table 3). However, in terms of healthcare resource utilization, the pattern of differences observed between the T2DM-raloxifene and non-DM-raloxifene groups (pre-matching) was similar to what was observed for the overall study group (pre- and post-matching).

**Table 3:** Comparison of fracture characteristics and healthcare resource utilization between groups (follow-up period; raloxifene subgroup)

	Unadjusted				Matched			
Characteristic	All (*N* = 1367)	T2DM (*N* = 668)	Non-DM (*N* = 699)	*P* value	All (*N* = 478)	T2DM (*N* = 239)	Non-DM (*N* = 239)	*P* value
Fractures								
Patients with clinical fractures^a^, n (%)	189 (13.8)	96 (14.4)	93 (13.3)	0.568	61 (12.8)	29 (12.1)	32 (13.4)	0.681
Number of days to first fracture, mean (SD)	440.8 (258.9)	398.2 (250.9)	484.8 (261)	0.499^b^	423.7 (248.7)	381.9 (244)	461.6 (250.6)	0.717^b^
Patients with non-vertebral fractures, n (%)	148 (10.8)	79 (11.8)	69 (9.9)	0.245	51 (10.7)	24 (10.0)	27 (11.3)	0.657
Resource utilization								
BMD test, n (%)	634 (46.4)	239 (35.8)	395 (56.5)	<0.001	232 (48.5)	96 (40.2)	136 (56.9)	<0.001
Bone formation test, n (%)	86 (6.3)	35 (5.2)	51 (7.3)	0.117	29 (6.1)	14 (5.9)	15 (6.3)	0.848
Bone resorption test, n (%)	157 (11.5)	60 (9.0)	97 (13.9)	0.005	54 (11.3)	26 (10.9)	28 (11.7)	0.773
Osteoporosis-related tests^c^ (post-baseline), n (%)	1029 (75.3)	488 (73.1)	541 (77.4)	0.063	364 (76.2)	178 (74.5)	186 (77.8)	0.391
Osteoporosis-related tests^c^ (post-baseline), mean per patient (SD)	2.863 (3.031)	2.867 (3.157)	2.86 (2.909)	0.966	2.747 (2.698)	2.816 (2.745)	2.678 (2.654)	0.576
Hospital admissions, n (%)	383 (28.0)	243 (36.4)	140 (20.0)	<0.001	129 (27.0)	79 (33.1)	50 (20.9)	0.003
Hospital admissions, mean per patient (SD)	0.49 (1.053)	0.668 (1.246)	0.32 (0.791)	<0.001	0.416 (0.849)	0.49 (0.839)	0.343 (0.855)	0.059
Days in hospital, mean (SD)	9.306 (29.13)	12.69 (35.26)	6.076 (21.23)	<0.001	8.073 (30.44)	9.586 (34.95)	6.561 (25.13)	0.278
Outpatient visits, n (%)	1367 (100.0)	668 (100.0)	699 (100.0)	-	478 (100.0)	239 (100.0)	239 (100.0)	-
Outpatient visits, mean per patient (SD)	32.72 (38.37)	38.61 (39.44)	27.09 (36.47)	<0.001	30.59 (34.09)	31.84 (23.96)	29.34 (41.85)	0.424
Laboratory tests, n (%)	268 (19.6)	162 (24.3)	106 (15.2)	<0.001	88 (18.4)	55 (23.0)	33 (13.8)	0.009
Laboratory tests, mean per patient (SD)	37.93 (127.3)	50.91 (151.9)	25.53 (96.77)	<0.001	27.54 (79.15)	36.03 (91.74)	19.05 (63.21)	0.019
Total cost in JPY, mean (SD)	750,000 (1,560,000)	1,010,000 (1,800,000)	500,000 (1,230,000)	<0.001	589,000 (1,170,000)	665,000 (980,000)	513,000 (1,340,000)	0.158
Total cost in JPY, median	240,000	396,000	144,000		215,000	314,000	150,000	<0.001^d^

*Abbreviations*: *BMD* bone mineral density, *DM* diabetes mellitus, *JPY* Japanese Yen, *SD* standard deviation, *T2DM* type 2 diabetes mellitus

^a^Clinical fractures include vertebral and non-vertebral fractures

^b^ *P* value from Log Rank test

^c^Osteoporosis-related tests include bone formation tests, bone resorption tests, imaging tests, and bone mineral density measurements

^d^ *P* value from Wilcoxon Rank Sum test

**Fig. 2:**
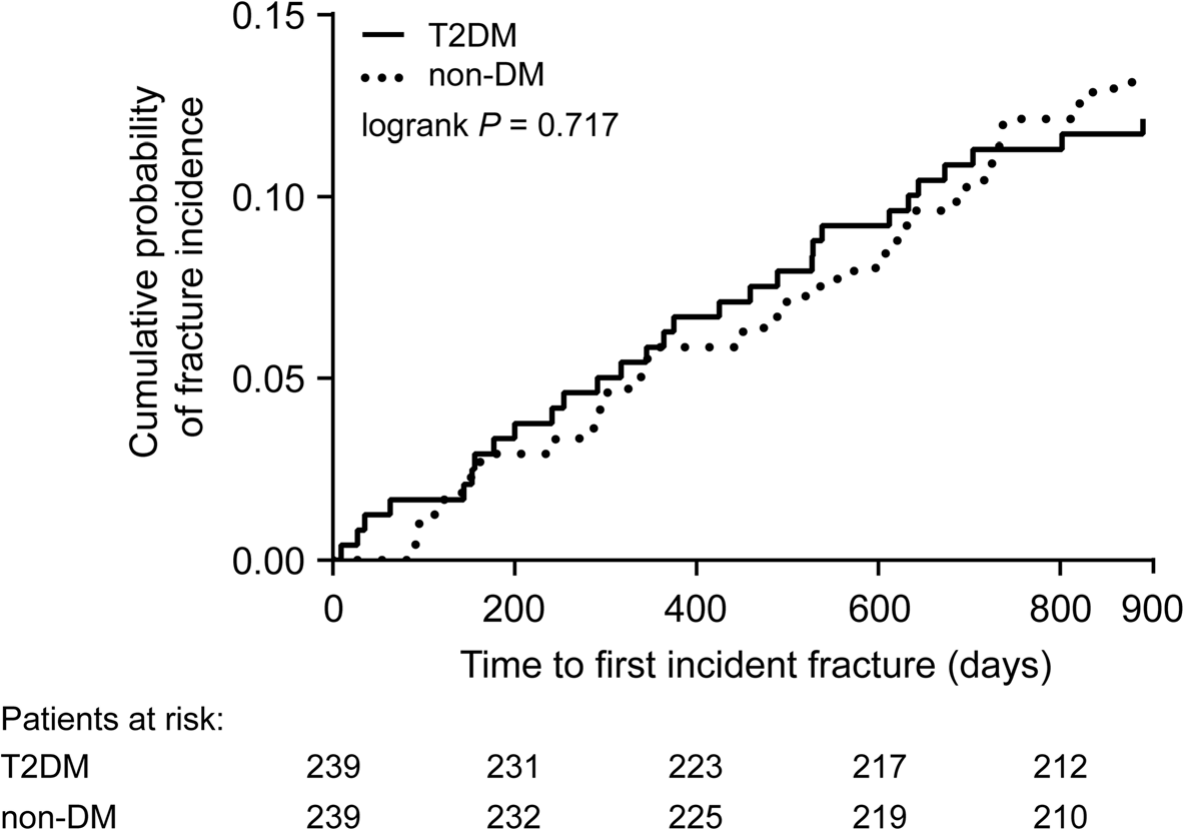
Kaplan-Meier curve of time to first incident fracture for the raloxifene subgroup, post-matching. Abbreviation: DM = diabetes mellitus; T2DM = type 2 diabetes mellitus

Following 1:1 exact matching, the number of significant differences between the T2DM and non-DM groups in the raloxifene subgroup was substantially reduced (Table 3). After matching, there were no differences in fracture characteristics or in the likelihood of fractures between groups (Table 3 and Fig. 2), consistent with what was observed before matching. The significant differences remaining after matching were in the percentages of patients with hospital admissions and laboratory tests (all of which were higher in the T2DM group), and the significantly lower percentage of patients in the T2DM group who underwent BMD tests.

Compared with the overall study group (post-matching; Table 2), after matching, the T2DM and non-DM groups in the raloxifene subgroup were more similar to each other (Table 3). In contrast to the overall study group, there were no significant differences in any of the fracture characteristics and there were no significant differences in the percentages of patients who underwent bone resorption tests or osteoporosis-related tests, the average number of outpatient visits per patient, or in the number of days spent in hospital.

## Discussion

To our knowledge, this is the first study to examine the real-world impact of T2DM on the incidence of fracture, cost, and healthcare resource utilization in patients with osteoporosis in Japan. As might be expected, at baseline, patients with T2DM incurred greater costs, utilized proportionally greater healthcare resources, and were in poorer health than patients without DM (Table 1). This is consistent with studies highlighting the considerable financial cost and resources required to adequately manage patients with T2DM, independent of osteoporosis [16, 17]. A similar pattern of differences in fracture characteristics and healthcare resource utilization was observed between the two groups following initiation of osteoporosis medication (during the follow-up period), even after 1:1 exact matching of baseline characteristics (Table 2). Although a proportion of patients in both the T2DM and non-DM groups developed clinical fractures during the follow-up period, the proportion in the T2DM group was significantly larger than in the non-DM group (12.7% vs 9.9%; *P* < 0.001; Table 2). This difference reflects the additional fracture risk posed by T2DM (independent of osteoporosis), as has been observed in previous studies [4, 5, 8]. These findings suggest that both physicians and patients need to carefully consider the potential impact of T2DM on the treatment and management of osteoporosis in order to achieve the best clinical outcome and to ensure the most efficient use of healthcare resources.

We used 1:1 exact matching to adjust for differences between the T2DM and non-DM groups, which resulted in the removal of most of the significant baseline differences between the two groups, indicating reduction of some of the existing bias. However, during the follow-up period, matching did little to change the pattern of significant differences observed between the two groups, indicating that these differences were likely to be real. Of note was the fact that the proportion of patients in the T2DM group who underwent BMD tests was significantly smaller than that in the non-DM group; this pattern was observed during follow-up (38.8% vs 44.5%; *P* < 0.001). This difference may be partly explained by the fact that BMD measurements have been suggested to be of limited use in predicting fracture risk in patients with chronic kidney disease (CKD), particularly in the later stages of CKD [18]. In fact, the 2009 Kidney Disease Improving Global Outcomes CKD-Mineral and Bone Disorder (CKD-MBD) Work Group does not recommend routine BMD testing to estimate fracture risk in patients with stage 3-5D CKD with evidence of CKD-MBD [19]. As the T2DM group had more patients with CKD, a lower rate of BMD testing amongst patients with CKD may have contributed to the significantly smaller proportion of patients undergoing BMD tests in the T2DM group compared with the non-DM group. Another explanation for the difference in BMD tests between the two groups may be that given the seriousness of the comorbidities and complications associated with diabetes (eg, nephropathy and retinopathy), patients diagnosed with T2DM may have prioritized management of T2DM in favor of osteoporosis [7], leading to fewer BMD tests being carried out. In Japan, patients with diabetes are often treated by endocrinologists, diabetologists, or internists, whereas patients with osteoporosis are often treated by orthopedic surgeons. This difference in medical care can lead to different treatment priorities, which may mean that patients with osteoporosis comorbid with diabetes may not receive optimal treatment for the increased fracture risk. The findings of this study highlight the need to educate both patients and physicians about the impact of diabetes on fracture risk so that screening and treatment of osteoporosis can be modified appropriately.

Compared with the overall study group, similar patterns in fracture characteristics and healthcare resource utilization were observed in the raloxifene subgroup, before matching (Table 3). However, in contrast to the overall study group, there were no statistically significant differences in any of the fracture characteristics between the T2DM and non-DM groups in the raloxifene subgroup during the 30 months of the follow-up period; this was observed in both unadjusted (clinical fracture: 14.4% vs 13.3%; *P* = 0.568) and matched groups (clinical fracture: 12.1% vs 13.4% *P* = 0.681). Following matching, there were even fewer significant differences between the two groups in terms of healthcare resource utilization. Taken together, these results may indicate that comorbid diabetes had a lower impact on fractures and healthcare resource utilization in patients who were prescribed raloxifene. Based on its mechanism of action, raloxifene has been proposed to affect diabetes-related fracture risk in a number of ways. Raloxifene may decrease the accumulation of advanced glycation endproducts, which are thought to reduce bone quality [11, 20]. Raloxifene has also been suggested to improve bone quality by increasing the formation of enzymatic cross-links in bone [13] and by improving collagen spacing [21] and bone toughness [22]. It is important to note that we cannot confirm that raloxifene alone is responsible for the beneficial effects observed or that these effects may not be mediated by other osteoporosis treatments. Furthermore, a recent study has suggested that the efficacy of bisphosophonates and raloxifene in reducing fracture risk is not affected by the presence of diabetes [23]. Nevertheless, our results indicate that further testing of raloxifene in this clinical setting, particularly in comparison with other osteoporosis medications, may be warranted.

In addition to considerations surrounding the efficacy of different osteoporosis treatments in patients with T2DM, physicians may also have to consider the safety profile of certain osteoporosis treatments in the context of diabetes. For instance, a retrospective analysis by Khamaisi and coauthors suggested that patients with diabetes may be at increased risk of developing osteonecrosis of the jaw when treated with bisphosphonates [24]. Conversely, physicians should also consider the effects of treatments for T2DM on osteoporosis. A meta-analysis of 10 randomized controlled trials suggested that long-term thiazolidinedione use doubles the risk of fractures among women with T2DM [25]. It is clear that comorbidity with T2DM adds further complexity to clinical decision-making during the treatment of osteoporosis.

The strengths of this study include the large number of patients and the fact that the analyses were performed on an extensive medical claims database, which reflects real-world clinical practice in more than 100 hospitals in Japan. Given that the data are drawn from a claims database, there are several inherent limitations, which include a reliance on ICD-10 codes being applied accurately and the fact that outcomes cannot be directly measured but must be inferred from available data. Although an exact matching method was used to minimize the differences between the T2DM and non-DM groups, there may have been additional unobserved confounding factors that were not considered during the matching process, which may have introduced bias into the comparisons. Additional limitations include the relatively short follow-up period, which may underestimate the incidence of fractures. Patients who were diagnosed with T2DM during the baseline period were not distinguished from those who were diagnosed during the follow-up period. The severity of osteoporosis in each patient was also not known; disproportionate numbers of patients with severe osteoporosis in each group may have introduced bias into the study. Patients may have also received their prescriptions from sources other than hospitals, which would not have been captured by the database utilized. Varying levels of adherence to and persistence with osteoporosis medications may have also affected the incidence of fractures observed. These limitations should be considered when interpreting the findings of this study and it should also be noted that no causative effects can be identified from this analysis.

## Conclusion

This study is one of the first to highlight the impact of T2DM on osteoporosis in real-world clinical practice in Japan. Patients with osteoporosis and T2DM had a higher incidence of fractures, utilized proportionally greater healthcare resources, and incurred proportionally higher healthcare costs, than matched patients without DM. This has important implications for clinical practice, as greater education of both patients and physicians may increase awareness of the substantial impact of diabetes on fracture risk in osteoporosis. In particular, physicians have to consider not only the increased fracture risk but the appropriateness of treatments for both osteoporosis and T2DM. Our analysis of study patients treated with raloxifene indicates that further assessment of this treatment in the context of T2DM may be warranted. Given the sizeable impact of osteoporosis on the economy and healthcare system of the “super-aging” society in Japan [26], strategies to offset the additional burden of T2DM on osteoporotic fractures may be of substantial value.

## Additional file

Additional file 1: Table S1. Clinical fractures (ICD-10 codes) included in the study. **Table S2.** Traumatic fractures (ICD-10 codes) excluded from the study. (DOCX 14 kb)
