# Supplementary material for: Fracture risk and healthcare resource utilization and costs among osteoporosis patients with type 2 diabetes mellitus and without diabetes mellitus in Japan: retrospective analysis of a hospital claims database
Source: BMC Musculoskelet Disord. 2016 Nov 25;17:489. doi: 10.1186/s12891-016-1344-9 (PMC5124298; doi:10.1186/s12891-016-1344-9)
Supplement: Additional file 1: Table S1. — Clinical fractures (ICD-10 codes) included in the study. Table S2. Traumatic fractures (ICD-10 codes) excluded from the study. (DOCX 14 kb) [file 12891_2016_1344_MOESM1_ESM.docx]

**Additional file 1**

**Table S1.** Clinical fractures (ICD-10 codes) included in the study

| Fracture Site | ICD-10 |
| --- | --- |
| Vertebral, pathological | M48.50XA, M80.08XA, M84.48XA, M84.68XA |
| Vertebral | S22.0, S32.0, S32.2, S12.9 |
|  |  |
| Hip, pathological | M84.459A, M84.359A |
| Hip | S72.0, S72.1, S72.2 |
|  |  |
| Wrist, pathological | M84.439A |
| Wrist/forearm | S52.0, S52.1, S52.2, S52.3, S52.5, S52.6, S52.9 |
|  |  |
| Humerus, pathological | M84.429A |
| Humerus | S42.2, S42.3, S42.4 |
|  |  |
| Clavicle/rib | S22.3,S22.4, S22.2, S22.0 |
|  |  |
| Pelvis, pathological | M84.350A |
| Pelvis | S32.3, S32.4, S32.5, S32.6, S32.8, S32.9 |
|  |  |
| Lower leg, pathological | M84.369A, M84.469A |
| Lower leg | S82.1, S82.2, S82.4, S82.1, S82.8,S82.3, S82.2 |
|  |  |
| Upper leg, pathological | M84.453A |
| Upper leg/femur | S72.3, S72.4, S72.9 |
|  |  |
| Other | S42.1, S62, S82.5, S82.6, S82.8, S92, T14.08, T07, M84.4 |

Abbreviations: ICD-10 = International Classification of Diseases, 10th Revision

**Table S2.** Traumatic fractures (ICD-10 codes) excluded from the study

| ICD-10 Injury code | Description |
| --- | --- |
| V00-V09 | Pedestrian injured in transport accident |
| V10-V19 | Pedal cycle rider injured in transport accident |
| V20-V29 | Motorcycle rider injured in transport accident |
| V30-V39 | Occupant of three-wheeled motor vehicle injured in transport accident |
| V40-V49 | Car occupant injured in transport accident |
| V50-V59 | Occupant of pick-up truck or van injured in transport accident |
| V60-V69 | Occupant of heavy transport vehicle injured in transport accident |
| V70-V79 | Bus occupant injured in transport accident |
| V80-V89 | Other land transport accidents |
| V90-V94 | Water transport accidents |
| V95-V97 | Air and space transport accidents |
| V98-V99 | Other and unspecified transport accidents |
| W13 | Fall from, out of or through building or structure |
| W14 | Fall from tree |
| W15 | Fall from cliff |
| W17 | Other fall from one level to another |
| W20-W49 | Exposure to inanimate mechanical forces |
| W50-W64 | Exposure to animate mechanical forces |
| W65-W74 | Accidental non-transport drowning and submersion |
| W85-W99 | Exposure to electric current, radiation and extreme ambient air temperature and pressure |
| X00-X08 | Exposure to smoke, fire and flames |
| X10-X19 | Contact with heat and hot substances |
| X30-X39 | Exposure to forces of nature |
| X52-X58 | Accidental exposure to other specified factors |
| X71-X83 | Intentional self-harm |
| X92-Y09 | Assault |
| Y21-Y33 | Event of undetermined intent |
| Y35-Y38 | Legal intervention, operations of war, military operations, and terrorism |
| Y62-Y69 | Misadventures to patients during surgical and medical care |
| Y70-Y82 | Medical devices associated with adverse incidents in diagnostic and therapeutic use |
| Y83-Y84 | Surgical and other medical procedures as the cause of abnormal reaction of the patient, or of later complication, without mention of misadventure at the time of the procedure |
| Y90-Y99 | Supplementary factors related to causes of morbidity classified elsewhere |

Abbreviations: ICD-10 = International Classification of Diseases, 10th Revision
